# Unintentional Injuries and Psychosocial Correlates among in-School Adolescents in Malaysia

**DOI:** 10.3390/ijerph121114936

**Published:** 2015-11-20

**Authors:** Karl Peltzer, Supa Pengpid

**Affiliations:** 1 ASEAN Institute for Health Development, Mahidol University, Salaya, Phutthamothon, Nakhon Pathom 73170, Thailand; E-Mail: supaprom@yahoo.com; 2 Department of Research & Innovation, University of Limpopo, Turfloop Campus, Sovenga 0727, South Africa; 3 Faculty of Public Health, Thammasat University, Klong Luang, Pathum Thani 12121, Thailand; 4 HIV/AIDS/STIs and TB (HAST), Human Sciences Research Council, Pretoria 0001, South Africa

**Keywords:** injury, psychosocial factors, substance use, secondary school children, Malaysia

## Abstract

The study aimed to provide estimates of the prevalence and psychosocial correlates of unintentional injury among school-going adolescents in Malaysia. Cross-sectional data from the Global School-Based Health Survey (GSHS) included 21,699 students (predominantly ≤13 to ≥17 years) that were selected by a two-stage cluster sample design to represent all secondary school students in Forms 1 to 5. The percentage of school children reporting one or more serious injuries in the past year was 34.9%, 42.1% of boys and 27.8% of girls. The two major causes of the most serious injury were “fall” (9.9%) and motor vehicle accident or being hit by a motor vehicle (5.4%), and the most frequent type of injury sustained was cut, puncture, or stab wound (6.2%) and a broken bone or dislocated joint (4.2%). In multivariable logistic regression analysis, sociodemographic factors (being male and low socioeconomic status), substance use (tobacco and cannabis use), frequent soft drink consumption, attending physical education classes three or more times a week, other risky behavior (truancy, ever having had sex, being bullied), psychological distress, and lack of parental or guardian bonding were associated with annual injury prevalence. Several factors were identified, which could be included in injury prevention promotion programs among secondary school children.

## 1. Introduction

Unintentional injuries are a major cause of disability and death among adolescents and youth, in particular in developing countries, including South Asia [1,2]. The unintentional injury of adolescents is an enormous public health problem [3]. The analysis of the prevalence of unintentional injury and potential risk factors can give possible insight into strategies of injury prevention and intervention [4]. There is a lack of data on the national injury prevalence and its psychosocial correlates among adolescents in Malaysia.

The prevalence of one or more serious injuries in the past year among adolescents in Asian countries was 19.7% in China [5], 45.9% in Indonesia [6], 20.3% in Iran [7], 27.0% in Myanmar [6], 37.2% in Sri Lanka [6], 27.3% in Taiwan [8], and 46.8% in Thailand [6]. In an adult community-based survey, the injury prevalence over the past two weeks was 3.1% in Malaysia [9]. The occurance of drowning in children (death and non-death) was estimated to be five per 100,000 in Malaysia [10], and the road traffic fatality rate in Malaysia in the age group (15–18 years old) was 3.9 per 100,000 population [11]. The two leading causes of hospital admissions in Malaysia due to unintentional injuries (0–19 years old) were traffic injuries and falls [12]. Major external causes of injury in various studies in Asian countries included “fall” [5,6,13], playing or training for a sport [6,7], vehicle accident or transport-related injuries [6,7,13], and burns [5,13].

Factors associated with unintentional injury among adolescents include: (1) sociodemographic factors, including male gender [6,7,14,15] and low socioeconomic status [6,16]; (2) psychological distress, including depressive prolems and sleep difficulty [6,15,17,18,19,20]; (3) behavioral and risk-taking behavior problems, including substance use (smoking and drinking alcohol), the experience of bullying, truancy, frequent participation in sport activities, and obesity [4,6,17,18,20,21]; and (4) home and school environment [15]. Some studies [22,23] have found an association between frequent soft drink consumption and violent behavior in adolescents, and it is possible that these adolescents may be more prone to sustaining injuries. The study aimed to provide estimates on the prevalence and psychosocial correlates of injury among secondary school students in Malaysia. Study findings could give possible insights for injury prevention strategies.

## 2. Methodology

### 2.1. Description of Survey and Study Population

This study was a secondary analysis of existing data from the Global School-Based Health Survey (GSHS) from Malaysia. GSHS details and data can be accessed [24]. The 2012 Malaysia GSHS used a two-stage (schools and classrooms) cluster sampling design to generate a nationally representative sample of students in Forms 1 to 5 (year 1 to 5 in secondary school) [25]. In all, 234 schools were systematically chosen “with a probability proportional enrollment using random start” [26], and classes were selected with “systematic equal probability sampling”, and from the selected classes all students were eligible to participate in this study [26]. Appropriate weights were used for inferences on all Grade 1 to 5 secondary school students in Malaysia [26]. The secondary school enrollment ratio was 69% in 2012 in Malaysia [27]. Students completed a self-administered questionnaire under the supervision of trained survey administrators during two classroom periods [25]. Parental consent was sought from all students from selected classes and non-consented students were registered as non-responses [25]. Approvals from both the “Ministry of Health Research and Ethics Committee” and the “Ministry of Education Ethics Committee” were obtained prior to the survey implementation [25].

### 2.2. Measures

The study variables used from the GSHS [24] are described in Table 1. Both the outcome variable, serious injury in the past year (1 = yes; 0 = no), and as well as independent variables were dichotomized. In addition, body weight and height were recorded by self-report, and obesity was classified as children with Body Mass Index (BMI) figures referring to an adult BMI of ≥30.0 kg/m^2^ using international age- and gender-specific criteria [28]. The GSHS questionnaire was found to have good validity in a previous validation study [29].

### 2.3. Data Analysis

Data analysis was conducted using STATA software version 12.0 (Stata Corporation, College Station, TX, USA). This software provides robust standard errors that account for the sampling design, *i.e.*, cluster sampling owing to the sampling of school classes. Associations between potential risk factors and injuries among school children, as largely identified from the literature review, were examined by calculating odds ratios (OR). Logistic regression was used for the examination of the impact of explanatory variables on the risk for injury (having had one or more serious injuries in the past 12 months = binary dependent variable). Potential multi-collinearity between variables was assessed with variance inflation factors, none of which exceeded the value of 1.6. Moreover, no statistical interaction (effect modification) was observed. Independent variables that significantly increased the injury risk in univariate analysis were included in the multivariable model. In reporting, weighted percentages are given, and the sample size reported reflects the sample that was asked the target question. The two-sided 95% confidence intervals are reported, and *p*-values less or equal to 5% are used to indicate statistical significance. The reported 95% confidence intervals and the *p*-values are both adjusted for the multi-stage stratified cluster sample design of the survey.

**Table 1:** Variable description.

Variables	Question	Response Options
Injury	During the past 12 months, how many times were you seriously injured? (An injury is serious when it makes you miss at least one full day of usual activities (such as school, sports, or a job) or requires treatment by a doctor or medical personnel.)	1 = 0 times 8 = 12 or more times
	During the past 12 months, what was the most serious injury that happened to you?	1 = I was not seriously injured during the past 12 months 2 = I had a broken bone or a dislocated joint, *etc*. See Table 2
	During the past 12 months, what was the major cause of the most serious injury that happened to you?	1 = I was not seriously injured during the past 12 months 2 = I was in a motor vehicle accident or hit by a motor vehicle, *etc*. See Table 2
Physical education	During this school year, on how many days did you go to physical education class (PE) each week?	1 = 0 days to 5 = 6 or more days
Soft drink consumption	During the past 30 days, how many times per day did you usually drink carbonated soft drinks such as Coca Cola, Sprite, and Pepsi? (Do not include diet soft drinks.)	1 = I did not drink carbonated soft drinks during the past 30 days 8 = 5 or more times per day
Current smoking cigarettes	During the past 30 days, on how many days did you smoke cigarettes?	1 = 0 days to 7 = All 30 days
Current other tobacco use	During the past 30 days, on how many days did you use tobacco products other than cigarettes such as shisha/hookah, electronic cigarettes, snuff, chewing tobacco, pipes, curut, cigars, cigarillos or bidis?	1 = 0 days to 7 = all 30 days
Current alcohol use	During the past 30 days, on how many days did you have at least one drink containing alcohol?	1 = 0 days to 7 = All 30 days
Lifetime cannabis use	During your life, how many times have you used marijuana?	1 = 0 times to 5 = 20 or more times
Truancy	During the past 30 days, on how many days did you miss classes or school without permission?	1 = 0 days to 10 or more days
Bullied	During the past 30 days, on how many days were you bullied?	1 = 0 days to 7 = All 30 days
Psychological distress	During the past 12 months, did you ever seriously consider attempting suicide?	1 = yes, 2 = no
	During the past 12 months, how often have you felt lonely?	1 = never to 5 = always
	How many close friends do you have?	1 = 0 to 4 = 3 or more
	During the past 12 months, how often have you been so worried about something that you could not sleep at night?	1 = never to 5 = always
Peer support	During the past 30 days, how often were most of the students in your school kind and helpful?	1 = never to 5 = always
Parental or guardian bonding	During the past 30 days, how often did your parents or guardians really know what you were doing with your free time?	1 = never to 5 = always

## 3. Results

### 3.1. Sample Characteristics

The overall response rate, a combination of school and student response rates, was 88.7% [25]. The final sample with complete injury data included 21,699 students. The distribution of the age, sex, and educational characteristics of the study sample are described in Table 2.

**Table 2:** Sample characteristics.

Variable	*n* = 21,699 (Unweighted Frequency)	% (Weighted Percent) (CI 95%)
**Sex**		
Male	10,640	50.5 (48.6–52.9)
Female	11,022	49.5 (47.6–51.4)
**Age**		
13 years or younger	4598	21.3 (20.0–23.0)
14 years	4603	20.7 (18.5–23.1)
15 years	4709	20.2 (18.6–21.9)
16 years	3850	19.0 (17.0–21.3)
17 years or older	3928	18.8 (16.7–20.9)
**School grade**		
Form 1	4488	21.1 (19.1–23.2)
Form 2	4574	20.5 (18.0–23.3)
Form 3	4756	20.0 (18.1–22.1)
Form 4	3827	19.2 (16.9–21.8)
Form 5	3871	18.3 (16.3–20.6)

### 3.2. Descriptive Results

The percentage of school children with one or more serious injuries in the past year was 34.9%, and that of those who had been injured more than once was 16.0%. The major cause of the most serious injury was “fall” (9.9%), followed by motor vehicle accident or being hit by a motor vehicle (5.4%), “something fell on me or hit me” (2.4%), and was attacked or abused or was fighting with someone (1.3%). Having been in a motor vehicle accident, falling and being attacked were significantly more frequent in boys than in girls. The most frequent type of injury sustained was “cut, puncture, or stab wound” (6.2%) and “a broken bone or dislocated joint” (4.2%) (see Table 3).

### 3.3. Associations with Injury Prevalence

In multivariable logistic regression analysis, sociodemographic factors (being male and hungery as indicators for low socioeconomic status), substance use (tobacco and cannabis use), frequent soft drink consumption, attending physical education classes three or more times a week, other risk behavior (truancy, ever having had sex,being bullied), psychological distress, and lack of parental or guardian bonding were associated with annual injury prevalence. In terms of main specific types of injuries, older age (16 or 17 years or older), being male, substance use (tobacco and cannabis), frequent soft drink consumption, truancy, and psychological distress were associated with motor vehicle-related injuries. Furthermore, younger age, being male, tobacco use, frequent soft drink consumption, frequent attendance of physical education classes, and other risk behaviors (truancy and being bullied) were associated with fall injuries (see Table 4).

**Table 3:** Annual prevalence of injury events, cause and type of injury by sex in percent (confidence interval 95%).

Variable	Total	Boys	Girls
**INJURY (in the past 12 months)**			
Injured once	34.9 (33.6–36.3)	42.1 (40.6–43.7)	27.8 (26.4–29.3)
Injured more than once	16.0 (15.2–16.9)	20.2 (19.1–21.4)	11.9 (11.0–12.8)
CAUSE (of most serious injury)			
I was in a motor vehicle accident or hit by a motor vehicle	5.4 (4.8–6.0)	7.5 (6.8–8.3)	3.2 (2.6–4.0)
**I fell**	9.9 (9.3–10.5)	11.9 (11.1–12.8)	7.8 (7.2–8.5)
Something fell on me or hit me	2.4 (2.2–2.7)	2.6 (2.2–3.1)	2.3 (2.0–2.6)
I was attacked or abused or was fighting with someone	1.3 (1.1–1.6)	2.0 (1.7–2.4)	0.7 (0.5–0.9)
I was in a fire or too near a flame or something hot	0.3 (0.2–0.4)	0.3 (0.2–0.5)	0.3 (0.2–0.4)
I inhaled or swallowed something bad for me	0.4 (0.3–0.6)	0.4 (0.3–0.6)	0.3 (0.2–0.6)
Something else caused my injury	7.1 (6.7–7.7)	7.9 (7.2–8.7)	6.4 (5.9–6.9)
**TYPE OF INJURY (of most serious injury)**			
I had a broken bone or a dislocated joint	4.2 (3.9–4.6)	6.3 (5.1–7.3)	2.4 (2.0–2.8)
I had a cut, puncture, or stab wound	6.2 (5.7–6.7)	8.3 (7.5–9.2)	4.9 (4.4–5.5)
I had a concussion or other head or neck injury, was knocked out, or could not breath	2.5 (2.3–2.8)	2.6 (2.2–3.0)	2.8 (2.4–3.2)
I had a gunshot wound	0.4 (0.3–0.5)	0.6 (0.5–0.9)	0.2 (0.16–0.4)
I had a bad burn	0.4 (0.3–0.5)	0.5 (0.4–0.7)	0.4 (0.2–0.6)
I was poisoned or took too much of a drug	0.2 (0.1–0.3)	0.2 (0.1–0.4)	0.2 (0.15–0.4)
**Something else happened to me**	9.4 (8.8–10.0)	11.3 (10.4–12.3)	8.7 (7.9–9.5)

**Table 4:** Logistic regression analysis for associations with injury (all injury types) and restricted analysis for motor vehicle and fall injuries.

	All Injuries		Motor Vehicle Injuries	Fall Injuries
	Odds Ratio (95% CI)	Odds Ratio (95% CI)	Odds Ratio (95% CI)	Odds Ratio (95% CI)
Variables	Crude	Adjusted	Adjusted	Adjusted
				
Age				
13 years or younger	1.00	1.00	1.00	1.00
14	1.01 (0.88–1.17)	1.00 (0.87–1.16)	1.06 (1.17–1.46)	1.07 (0.86–1.32)
15	0.94 (0.78–1.13)	0.97 (0.81–1.16)	1.26 (0.91–1.75)	0.99 (0.79–1.23)
16	0.90 (0.77–1.04)	0.90 (0.77–1.05)	1.49 (1.13–1.95) **	0.79 (0.64–0.99) *
17 years or older	0.84 (0.72–0.98) *	0.88 (0.75–1.03)	1.58 (1.17–2.13) **	0.63 (0.51–0.79) ***
Gender				
Female	1.00	1.00	1.00	1.00
Male	1.89 (1.76–2.03) ***	1.56 (1.44–1.70) ***	1.61 (1.24–2.08) ***	1.53 (1.34–1.76) ***
Hunger (4.7%)	2.16 (1.86–2.50) ***	1.44 (1.19–1.75) ***	0.98 (0.67–1.45)	1.04 (0.75–1.44)
Current any tobacco use (12.5%)	3.00 (2.03–3.41) ***	1.86 (1.64–2.11) ***	3.48 (2.83–4.26) ***	1.26 (1.05–1.50) *
Current drinking (8.7%)	1.76 (1.57–1.97) ***	1.02 (0.88–1.17)	0.91 (0.66–1.28)	0.98 (0.80–1.20)
Ever cannabis use (0.9%)	10.42 (6.77–16.04) ***	2.72 (1.63–4.53) ***	2.15 (1.19–3.87) *	0.82 (0.39–2.00)
Soft drink (1 plus/day) (29.2%)	1.83 (1.71–1.95) ***	1.44 (1.31–1.57) ***	1.36 (1.10–1.87) **	1.25 (1.09–1.42) ***
Physical education classes 3 or more times a week (26.0%)	1.14 (1.05–1.14) **	1.12 (1.02–1.22) *	1.00 (0.84–1.19)	1.25 (1.08–1.44) **
Truancy in the past month (30.6%)	1.80 (1.66–1.95) ***	1.45 (1.32–1.60) ***	1.73 (1.44–2.08) ***	1.37 (1.19–1.57) ***
Ever sex (8.2%)	1.92 (1.67–2.22) ***	1.28 (1.07–1.53) **	1.30 (0.97–1.73)	0.84 (0.64–1.09)
Bullied in the past month (17.3%)	2.95 (261–3.33) ***	2.15 (1.88–2.45) ***	1.21 (0.96–1.52)	1.70 (1.48–1.96) ***
Obesity (9.6%)	0.99 (0.88–1.12)	---	1.02 (0.77–1.35)	1.04 (0.85–1.27)
Psychological distress				
Zero (81.9%)	1.00	1.00	1.00	1.00
One (13.8%)	1.59 (1.43–1.77) ***	1.43 (1.25–1.64) ***	1.40 (1.07–1.83) *	1.13 (0.93–1.38)
Two or more (4.4%)	2.76 (2.28–3.34) ***	2.04 (1.59–2.61) ***	1.39 (0.85–2.26)	1.21 (0.90–1.61)
Peer support (mostly or always) (44.8%)	0.76 (0.71–0.81) ***	1.01 (0.94–1.10)	0.81 (0.64–1.02)	1.10 (0.96–1.26)
Parental or guardian bonding (mostly or always) (43.3%)	0.72 (0.66–0.77) ***	0.91 (0.84–0.98) *	1.12 (0.94–1.33)	0.97 (0.85–1.11)

CI = Confidence interval; *** *p* < 0.000; ** *p* < 0.01; * *p* < 0.05.

## 4. Discussion

In this investigation of a large sample of secondary school-going adolescents in Malaysia, an annual injury prevalence of 34.9% was found, which is higher than in a few previous studies in Asian countries including China, Iran, Myanmar, and Taiwan [5,6,8], similar to Sri Lanka [6], and lower than what was found in Indonesia and Thailand [6]. Regarding the cause of the injury, the study found that the highest annual prevalence rate was in falling and motor vehicle-related injuries, which was similar to findings from various studies in Asian countries [5,6,7,13]. Hyder *et al.* [30] note that “the overall average incidence rate for childhood falls is highest in South America at 1315 followed by Asia at 1036 per 100,000, respectively”. The most frequent type of injury sustained in this study was “cut, puncture, or stab wound” (6.2%) and “a broken bone or dislocated joint” (4.2%). In a previous GSHS in four Southeast Asian countries, the prevalence of broken bones or dislocated joints was 10.1%, while cut, puncture, or stab wounds was 5.1% [6].

In agreement with previous studies [6,7,14,15], male gender was found in this study to be associated with injury, in particular having been in a motor vehicle-related accident, falling, and being attacked, while for other types of injury such as “something fell on me or hit me”, “I was in a fire or too near a flame or something hot”, and “I inhaled or swallowed something bad for me”, no sex differences were found. Further, hunger as a variable representing low socioeconomic status was, in this survey, associated with annual injury prevalence, as also found in previous studies [6,16]. However, the socioeconomic association was not observed for specific injury outcomes (falls and motor vehicle-related injury), which may be because of the limited number of cases in these subgroups. It is possible that children from lower socioeconomic backgrounds have greater material deprivation and societal barriers to being protected from injury [31].

Moreover, psychological distress was, in this study in multivariate analysis, significantly associated with injury. This finding was confirmed in previous studies [6,15,17,18,19,20]. Substance use (tobacco and cannabis) was, as found in some previous studies [6,17,18], associated with injury in this adolescent population. Elevated psychological distress and substance use seem to play a role in adolescent injury, which calls for interventions to incorporate the minimization of poor mental health in adolescent injury prevention [32].

Although, in general, there were no age differences in relation to annual injury prevalence, there were age differences regarding specific types of causes of injuries; older age (16 or 17 years or older) was associated with a motor vehicle-related injury and younger students had a higher annual prevalence of falls than older students. The study found that attending physical education classes three or more times a week was associated with overall injury and fall injuries, in particular. Previous studies found that frequent participation in sport activities [6,17,18] was associated with sustaining injuries.

The study found that other risk behaviors (truancy, ever having had sex, being bullied) were associated with injury, which is partly confirmed in other studies [6,17,18]. Some previous studies (e.g., [15]) had identified an association between home and school environment and injury risk, while this survey found that a lack of parental or guardian bonding was related to annual injury prevalence. Contrary to one previous study [20], obesity was not found to be associated with annual injury prevalence. Further, frequent soft drink consumption was associated with higher odds of injury in this survey. Solnick and Hemenway [22] found, in US adolescents, a strong association between frequent “carbonated non-diet soft drink consumption” and having carried a weapon, having been violent with peers, dates, and family members, and involvement in physical fighting [23]. Therefore, it may be possible that this increase in the probability of engaging in aggressive actions may result in more injuries.

Overall, the study found, as in some previous studies [6,17], that the odds for all injuries increased with an increase in the number of psychological distresses and other risk behaviors such as substance use, truancy, being bullied, ever having had sex, and frequent soft drink consumption. Therefore, it may be indicative to target multiple types of risk behaviors, including injury, simultaneously in health promotion interventions [33].

This analysis represents one of the first national examinations of adolescent injury in Malaysia. Injuries sustained outside the home *vs*. those sustained in the home as well as injuries that were inflicted by others *vs*. those that were self-inflicted were not assessed in the Malaysian GSHS. Depending on, for example, the location of where most injuries had occurred, such as at school, at home, or on the road, interventions could be more specifically targeted [34].

### Study Limitations

The study focused on secondary school-going adolescents who may not be representative of all adolescents in Malaysia as the prevalence of injury events and associated factors may be different between school-going and non-school-going adolescents. Further, the questionnaire used was completed by self-report, which may have introduced a reporting bias. Moreover, this study was based on data collected in a cross-sectional survey, and we can therefore not ascribe causality to any of the associated factors in the study. The analysis was limited to the variables which had been included in the GSHS in Malaysia, and other additional risk factors and the location of the injury could have been included [6]. Another limitation was that some concepts such as peer support were assessed in this study with single items, and future studies should use scales. Finally, there may be an underestimation of the annual injury prevalence in this study because the questionnaire used only collected information on the most serious injury and over a long (12 month) recall period.

## 5. Conclusions

In this study, a high annual prevalence of injury was found among a large sample of school-going adolescents in Malaysia. Various associations with injury were identified, including psychological distresses, substance use, truancy, being bullied, ever having had sex, and frequent soft drink consumption, which may be considered in an integrated approach to injury prevention and safety promotion strategies among school children.
